# Appropriation for the John Hopkins University

**Published:** 1899-04

**Authors:** 


The Maryland State Legislature has appropriated $400,000 to the John Hopkins University to tide it over its financial straits, due to the loss sustained in the default of the Baltimore & Ohio Railroad.

